# 6,6′-(Pyridine-2,6-di­yl)bis­(pyrrolo­[3,4-*b*]pyridine-5,7-dione)

**DOI:** 10.1107/S160053681104414X

**Published:** 2011-10-29

**Authors:** P. C. W. Van der Berg, Hendrik G. Visser, Andreas Roodt

**Affiliations:** a Department of Chemistry, University of the Free State, PO Box 339, Bloemfontein 9300, South Africa

## Abstract

The title compound, C_19_H_9_N_5_O_4_, has crystallographically imposed twofold rotational symmetry. The asymmetric unit contains one half-mol­ecule. The crystal structure is stabilized by π–π stacking of inversion-related pyrrolo­[3,4-*b*]pyridine rings, with a centroid–centroid distance between stacked pyridines of 3.6960 (8) Å. The dihedral angle between the central pyridine ring and the pyrrolo-pyridine side rings is 77.86 (2)° while the angle between the two side chains is 60.87 (2)°.

## Related literature

For related structures, see: Jain *et al.* (2004 ▶). For related metal complexes, see: Schutte *et al.* (2009 ▶, 2010 ▶); Brink *et al.* (2011 ▶).
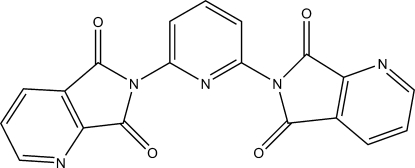


## Experimental

### 

#### Crystal data

C_19_H_9_N_5_O_4_
*M* *_r_* = 371.31
Monoclinic, 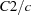

*a* = 14.539 (1) Å
*b* = 7.391 (1) Å
*c* = 15.686 (1) Å
β = 108.752 (2)°
*V* = 1596.1 (3) Å^3^
*Z* = 4
Mo *K*α radiation
μ = 0.11 mm^−1^
*T* = 100 K
0.34 × 0.29 × 0.27 mm

#### Data collection

Bruker X8 APEXII 4K KappaCCD diffractometer
Absorption correction: multi-scan (*SADABS*; Bruker, 2004 ▶) *T*_min_ = 0.681, *T*_max_ = 0.746
12803 measured reflections
1920 independent reflections
1717 reflections with *I* > 2σ(*I*)
*R*_int_ = 0.024

#### Refinement

*R*[*F* ^2^ > 2σ(*F* ^2^)] = 0.034
*wR*(*F* ^2^) = 0.091
*S* = 1.06
1920 reflections
128 parameters
H-atom parameters constrained
Δρ_max_ = 0.31 e Å^−3^
Δρ_min_ = −0.21 e Å^−3^

### 

Data collection: *APEX2* (Bruker, 2010 ▶); cell refinement: *SAINT-Plus* (Bruker, 2004 ▶); data reduction: *SAINT-Plus*; program(s) used to solve structure: *SHELXS97* (Sheldrick, 2008 ▶); program(s) used to refine structure: *SHELXL97* (Sheldrick, 2008 ▶); molecular graphics: *DIAMOND* (Brandenburg & Putz, 2005 ▶); software used to prepare material for publication: *WinGX* (Farrugia, 1999 ▶).

## Supplementary Material

Crystal structure: contains datablock(s) global, I. DOI: 10.1107/S160053681104414X/pk2352sup1.cif

Structure factors: contains datablock(s) I. DOI: 10.1107/S160053681104414X/pk2352Isup2.hkl

Supplementary material file. DOI: 10.1107/S160053681104414X/pk2352Isup3.cml

Additional supplementary materials:  crystallographic information; 3D view; checkCIF report

## Figures and Tables

**Fig. 1.**
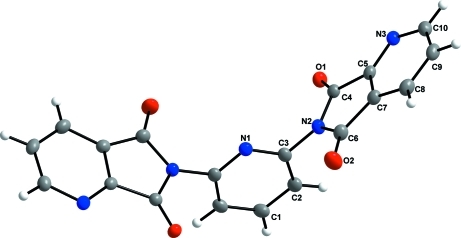
Molecular structure of the title compound. Displacement ellipsoids are drawn at the 50% probability level. Unlabelled atoms are related to their labelled counterparts by a crystallographic 2-fold rotation about b.

**Fig. 2.**
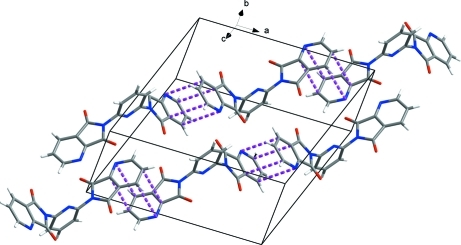
Packing and illustration of π-π stacking in the crystal.

## supplementary crystallographic information

### Comment

The title compound was synthesized as a ligand for potential use in medical and radiopharmaceutical applications (Schutte *et al.*, 2009; Schutte *et al.*, 2010; Brink *et al.*, 2011).

The title compound, C_19_H_9_N_5_O_4_, has crystallographically imposed two-fold rotational symmetry.  The asymmetric unit contains one half-molecule with C1, H1 and N1 lying on a two-fold rotational axis. The dihedral angle between the central pyridine ring and the pyrrolo-pyridine side rings is 77.86 (2)° while the angle between the two side chains is 60.87 (2)°.

In the crystal, all bond distances and angles are normal (Jain *et al.* (2004).  The molecules pack in layers, diagonally across the *ac* plane in a head-to-tail fashion and the structure is stabilized by π-π stacking between the outlying pyridine rings of inversion-related structures.  The centroid to centroid distances between these stacked rings = 3.6960 (8) Å (see Fig. 2).

### Experimental

Under oxygen atmosphere: 2,3-pyridinedicarboxylic acid (1.000 g, 5.982 mmol) was added as a solid in one portion to a suspension of 2,6-diaminopyridine (0.3092 g, 2.833 mmol) in pyridine (10 ml) and the mixture was stirred at 40 °C for 40 min. Triphenylphosphite (10 ml) was added dropwise over 10 minutes after which the temperature was increased to 90–100 °C and stirred for a further 24 h. On cooling the precipitate was filtered, washed with H_2_O (50 ml) and then MeOH (50 ml). The precipitate was recrystallized in chloroform to obtain colourless crystals after five days.

### Refinement

The aromatic H atoms were placed in geometrically idealized positions at C—H = 0.93 Å, respectively and constrained to ride on their parent atoms, with *U*_iso_(H) = 1.2*U*_eq_(C). The highest peak is located 0.67 Å from C5 and the deepest hole is situated 1.26 Å from C1

### Crystal data

**Table:**

C_19_H_9_N_5_O_4_	*F*(000) = 760
*M**_r_* = 371.31	*D*_x_ = 1.545 Mg m^−^^3^
Monoclinic, *C*2/*c*	Mo *K*α radiation, λ = 0.71073 Å
*a* = 14.539 (1) Å	Cell parameters from 6738 reflections
*b* = 7.391 (1) Å	θ = 2.7–28.3°
*c* = 15.686 (1) Å	µ = 0.11 mm^−^^1^
β = 108.752 (2)°	*T* = 100 K
*V* = 1596.1 (3) Å^3^	Cuboid, colourless
*Z* = 4	0.34 × 0.29 × 0.27 mm

### Data collection

**Table:**

Bruker X8 APEXII 4K KappaCCD diffractometer	1920 independent reflections
Radiation source: fine-focus sealed tube	1717 reflections with *I* > 2σ(*I*)
graphite	*R*_int_ = 0.024
ω and φ scans	θ_max_ = 28°, θ_min_ = 3.1°
Absorption correction: multi-scan (*SADABS*; Bruker, 2004)	*h* = −19→15
*T*_min_ = 0.681, *T*_max_ = 0.746	*k* = −9→9
12803 measured reflections	*l* = −20→20

### Refinement

**Table:**

Refinement on *F*^2^	Primary atom site location: structure-invariant direct methods
Least-squares matrix: full	Secondary atom site location: difference Fourier map
*R*[*F*^2^ > 2σ(*F*^2^)] = 0.034	Hydrogen site location: inferred from neighbouring sites
*wR*(*F*^2^) = 0.091	H-atom parameters constrained
*S* = 1.06	*w* = 1/[σ^2^(*F*_o_^2^) + (0.0432*P*)^2^ + 1.2599*P*] where *P* = (*F*_o_^2^ + 2*F*_c_^2^)/3
1920 reflections	(Δ/σ)_max_ < 0.001
128 parameters	Δρ_max_ = 0.31 e Å^−^^3^
0 restraints	Δρ_min_ = −0.21 e Å^−^^3^

### Special details

**Table:**

Experimental. The intensity data were collected on a Bruker X8 ApexII 4 K Kappa CCD diffractometer using an exposure time of 30 s/frame. A total of 1758 frames were collected with a frame width of 0.5° covering up to θ = 28.00° with 99.3% completeness accomplished.
Geometry. All s.u.'s (except the s.u. in the dihedral angle between two l.s. planes) are estimated using the full covariance matrix. The cell s.u.'s are taken into account individually in the estimation of s.u.'s in distances, angles and torsion angles; correlations between s.u.'s in cell parameters are only used when they are defined by crystal symmetry. An approximate (isotropic) treatment of cell s.u.'s is used for estimating s.u.'s involving l.s. planes.
Refinement. Refinement of *F*^2^ against ALL reflections. The weighted *R*-factor *wR* and goodness of fit *S* are based on *F*^2^, conventional *R*-factors *R* are based on *F*, with *F* set to zero for negative *F*^2^. The threshold expression of *F*^2^ > 2σ(*F*^2^) is used only for calculating *R*-factors(gt) *etc*. and is not relevant to the choice of reflections for refinement. *R*-factors based on *F*^2^ are statistically about twice as large as those based on *F*, and *R*- factors based on ALL data will be even larger.

### Fractional atomic coordinates and isotropic or equivalent isotropic displacement parameters (Å^2^)

**Table:**

	*x*	*y*	*z*	*U*_iso_*/*U*_eq_	
O1	−0.05122 (6)	0.18475 (11)	0.00789 (5)	0.0226 (2)	
O2	0.22325 (6)	0.31769 (14)	0.24267 (6)	0.0327 (2)	
N1	0	0.22247 (19)	0.25	0.0206 (3)	
N2	0.07684 (7)	0.22769 (13)	0.13958 (6)	0.0209 (2)	
N3	0.07688 (7)	0.39842 (13)	−0.07379 (6)	0.0212 (2)	
C7	0.17897 (8)	0.39008 (15)	0.08242 (7)	0.0210 (2)	
C4	0.02795 (8)	0.24566 (15)	0.04720 (7)	0.0186 (2)	
C10	0.15034 (8)	0.48687 (16)	−0.09009 (8)	0.0231 (2)	
H10	0.1419	0.5224	−0.149	0.028*	
C6	0.16835 (8)	0.31349 (16)	0.16665 (8)	0.0232 (2)	
C5	0.09539 (8)	0.35189 (15)	0.01192 (7)	0.0184 (2)	
C8	0.25407 (8)	0.48175 (16)	0.06507 (8)	0.0249 (3)	
H8	0.3115	0.5102	0.1104	0.03*	
C9	0.23809 (8)	0.52873 (16)	−0.02460 (8)	0.0246 (3)	
H9	0.2863	0.5884	−0.0407	0.029*	
C3	0.03783 (8)	0.12612 (16)	0.19763 (7)	0.0205 (2)	
C2	0.04021 (8)	−0.06106 (16)	0.19451 (7)	0.0228 (2)	
H2	0.0678	−0.1208	0.1565	0.027*	
C1	0	−0.1562 (2)	0.25	0.0237 (3)	
H1	0	−0.282	0.25	0.028*	

### Atomic displacement parameters (Å^2^)

**Table:**

	*U*^11^	*U*^22^	*U*^33^	*U*^12^	*U*^13^	*U*^23^
O1	0.0175 (4)	0.0269 (4)	0.0224 (4)	−0.0041 (3)	0.0052 (3)	−0.0019 (3)
O2	0.0241 (5)	0.0474 (6)	0.0219 (4)	−0.0071 (4)	0.0010 (4)	−0.0015 (4)
N1	0.0173 (6)	0.0254 (7)	0.0177 (6)	0	0.0037 (5)	0
N2	0.0179 (5)	0.0265 (5)	0.0180 (4)	−0.0026 (4)	0.0053 (4)	−0.0012 (4)
N3	0.0210 (5)	0.0210 (5)	0.0226 (5)	0.0000 (4)	0.0084 (4)	−0.0002 (4)
C7	0.0184 (5)	0.0217 (5)	0.0223 (5)	0.0000 (4)	0.0059 (4)	−0.0032 (4)
C4	0.0182 (5)	0.0193 (5)	0.0187 (5)	0.0010 (4)	0.0064 (4)	−0.0017 (4)
C10	0.0250 (6)	0.0208 (5)	0.0262 (5)	0.0007 (4)	0.0120 (5)	0.0008 (4)
C6	0.0189 (5)	0.0268 (6)	0.0230 (5)	−0.0019 (4)	0.0053 (4)	−0.0033 (4)
C5	0.0162 (5)	0.0176 (5)	0.0221 (5)	0.0005 (4)	0.0070 (4)	−0.0027 (4)
C8	0.0177 (5)	0.0251 (6)	0.0306 (6)	−0.0027 (4)	0.0062 (5)	−0.0037 (5)
C9	0.0214 (5)	0.0206 (5)	0.0352 (6)	−0.0021 (4)	0.0141 (5)	−0.0011 (5)
C3	0.0168 (5)	0.0271 (6)	0.0162 (5)	−0.0012 (4)	0.0033 (4)	−0.0001 (4)
C2	0.0227 (5)	0.0272 (6)	0.0168 (5)	0.0012 (4)	0.0043 (4)	−0.0023 (4)
C1	0.0273 (8)	0.0232 (8)	0.0179 (7)	0	0.0036 (6)	0

### Geometric parameters (Å, °)

**Table:**

O1—C4	1.2047 (13)	C4—C5	1.4944 (15)
O2—C6	1.2033 (14)	C10—C9	1.3915 (17)
N1—C3	1.3322 (13)	C10—H10	0.93
N1—C3^i^	1.3322 (13)	C8—C9	1.3938 (17)
N2—C4	1.4001 (14)	C8—H8	0.93
N2—C6	1.4105 (14)	C9—H9	0.93
N2—C3	1.4306 (14)	C3—C2	1.3851 (17)
N3—C5	1.3286 (14)	C2—C1	1.3860 (14)
N3—C10	1.3450 (15)	C2—H2	0.93
C7—C5	1.3840 (15)	C1—C2^i^	1.3860 (14)
C7—C8	1.3843 (16)	C1—H1	0.93
C7—C6	1.4904 (16)		
			
C3—N1—C3^i^	115.37 (14)	N3—C5—C4	124.55 (10)
C4—N2—C6	112.63 (9)	C7—C5—C4	108.86 (9)
C4—N2—C3	122.37 (9)	C7—C8—C9	115.73 (11)
C6—N2—C3	124.95 (9)	C7—C8—H8	122.1
C5—N3—C10	113.78 (10)	C9—C8—H8	122.1
C5—C7—C8	119.22 (10)	C10—C9—C8	120.36 (11)
C5—C7—C6	108.44 (10)	C10—C9—H9	119.8
C8—C7—C6	132.32 (10)	C8—C9—H9	119.8
O1—C4—N2	125.30 (10)	N1—C3—C2	125.15 (11)
O1—C4—C5	129.75 (10)	N1—C3—N2	116.02 (10)
N2—C4—C5	104.95 (9)	C2—C3—N2	118.82 (10)
N3—C10—C9	124.28 (11)	C3—C2—C1	117.65 (11)
N3—C10—H10	117.9	C3—C2—H2	121.2
C9—C10—H10	117.9	C1—C2—H2	121.2
O2—C6—N2	124.82 (11)	C2—C1—C2^i^	119.04 (16)
O2—C6—C7	130.09 (11)	C2—C1—H1	120.5
N2—C6—C7	105.09 (9)	C2^i^—C1—H1	120.5
N3—C5—C7	126.59 (10)		

Symmetry codes: (i) −*x*, *y*, −*z*+1/2.
